# High diagnostic accuracy of quantitative SARS-CoV-2 spike-binding-IgG assay and correlation with *in vitro* viral neutralizing activity

**DOI:** 10.1016/j.heliyon.2024.e24513

**Published:** 2024-01-13

**Authors:** Noriko Iwamoto, Yuki Takamatsu, Yusuke Asai, Kiyoto Tsuchiya, Kouki Matsuda, Yusuke Oshiro, Natsumi Inamura, Mari Terada, Takashi Nemoto, Moto Kimura, Sho Saito, Shinichiro Morioka, Maeda Kenji, Hiroaki Mitsuya, Norio Ohmagari

**Affiliations:** a Department of Disease Control Center, Center Hospital of the National Center for Global Health and Medicine, Tokyo, Japan; b Refractory Viral Diseases, National Center for Global Health and Medicine Research Institute, Tokyo, Japan; c AIDS Clinical Center, Center Hospital of the National Center for Global Health and Medicine, Tokyo, Japan; d Clinical Laboratory Department, Center Hospital of the National Center for Global Health and Medicine, Tokyo, Japan; e Center for Clinical Sciences, National Center for Global Health and Medicine, Tokyo, Japan; f Division of Antiviral Therapy, Joint Research Center for Human Retrovirus Infection, Kagoshima University, Japan; g Experimental Retrovirology Section, Center for Cancer Research, National Cancer Institute, National Institutes of Health, Bethesda, MD, USA; h Department of Clinical Sciences, Kumamoto University School of Medicine, Kumamoto, Japan

**Keywords:** COVID-19, SARS-CoV-2, Quantitative anti-SARS-CoV-2-spike-IgG antibody test, Neutralizing antibody, Diagnostic tools

## Abstract

**Background:**

Antibody testing can easily evaluate the clinical status of patients, aid in the diagnosis of multisystem inflammatory syndrome, and monitor the immunity level in the population. However, the applicability of serological tests in detecting antibodies against the severe acute respiratory syndrome 2 (SARS-CoV-2) spike-binding protein remains limited. This study aimed to quantify both serum-derived neutralizing immunoglobulin-G (IgG) antibody activity and the amount of anti-SARS-CoV-2 Spike-IgG (S-IgG) in convalescent sera/plasmas and evaluate the direct correlation between the *in vitro* IgG-EC__50__ values and S-IgG values.

**Methods:**

We evaluated the neutralizing activity of purified IgG (IgG-EC_50_), quantified S-IgG in the serum/plasma of consecutive COVID-19 convalescent individuals using a cell-based virus-neutralizing assay, and determined the correlation between IgG-EC_50_ and S-IgG. In addition, we evaluated rational cut-off values using the receiver operating characteristic (ROC) curve and calculated the sensitivity and specificity of the quantitative S-IgG assay for moderate and high IgG-EC_50_.

**Results:**

A high correlation was observed between S-IgG and IgG-EC_50_ with a Spearman's ρ value of −0.748 (95 % confidence interval [CI]: −0.804–0.678). Using an IgG-EC_50_ of 50 μg/mL and 20 μg/mL as the cut-off values for moderate and high *in vitro* neutralizing activity, respectively, the Youden's index values of 287.5 binding antibody units (BAU)/mL and 454.1 BAU/mL determined from the ROC curve showed the highest diagnostic accuracy, with Kappa values of 0.884 (95 % CI: 0.823–0.946) and 0.920 (95 % CI: 0.681–0.979), respectively.

**Conclusions:**

Quantitative S-IgG tests are a useful and convenient tool for estimating *in vitro* virus-neutralizing activity, with a high correlation with IgG-EC_50_ when the rational cut-off value is carefully determined.

## Highlights

• Quantified SARS-CoV-2 Spike-binding-IgG correlates with neutralizing activity.
• Rational cut-off values enhance the diagnostic accuracy of serological tests.
• Quantitative anti-SARS-CoV-2 Spike-immunoglobulin G test is useful diagnostically.
• COVID 19-convalescent individuals possess serum virus-neutralizing activity.

## 1 Introduction

Coronavirus disease 2019 (COVID-19) convalescent patients and individuals who received COVID-19 vaccines developed specific immune responses against severe acute respiratory syndrome coronavirus 2 (SARS-CoV-2) following exposure to its antigens. Before the emergence of the Omicron variant (PANGO lineage B.1.1.529 or BA.*) of the SARS-CoV-2, studies suggested that immunoglobulin G (IgG) antibodies targeting viral proteins played a crucial role in reducing the risk of re-infection [[1], [2], [3], [4], [5]]. However, it remains uncertain to what degree and for how long these antibodies may protect against symptomatic infection, especially with the emergence of variants with multiple amino acid mutations.

Serological antibody tests are one of the easiest methods to verify the presence of anti-pathogenic antibodies after an infection or vaccination. Regarding COVID-19, serum neutralizing activity is highly correlated with the amount of anti-SARS-CoV-2 Spike-IgG (S-IgG) antibodies in the serum, especially the receptor-binding domain (RBD)-binding antibodies [6,7]. As of March 2023, many semi-quantitative antibody tests have received the Emergency Use Authorization from the United States Food and Drug Administration (FDA) [8]. Antibody testing can easily evaluate the clinical status (whether the person had past infection or vaccination), aid in the diagnosis of multisystem inflammatory syndrome in children and adults, and monitor the levels of immunity *(e.g.*, infection rate, vaccination rate) in the population. On the other hand, it has not been recommended to assess the degree of immunity to SARS-CoV-2 or the need for vaccination so far, although the amount of virus-specific antibodies is expectedly related to the level of disease protection [9,10]. Thus, an advanced assessment method that proves the direct correlation between the amount and activity of the neutralizing IgG antibodies with a screening or diagnostic test, especially based on the World Health Organization international standard for anti-SARS-CoV-2-immunoglobulins [11], is needed.

In this community-based cohort study, we aimed to retrospectively evaluate the neutralizing activity of serum/plasma-derived purified IgG *in vitro* using a cell-based virus-neutralizing assay and identify S-IgG using the chemiluminescence microparticle immunoassay. Moreover, we reported a direct, high correlation between the level of S-IgG and the neutralizing activity of the purified IgG (IgG-EC_50_), and the rational cut-off value was carefully determined. we sought to address the gap between the clinical need for antibody testing and the limitation of the assay system. Thus, this study aimed to quantify both serum-derived neutralizing IgG antibody activity and the amount of S-IgG using approximately 200 convalescent sera/plasmas and evaluate the direct correlation between the *in vitro* IgG-EC_50_ values and S-IgG values. Further, we generated receiver operating characteristic (ROC) curves using three different cut-off values, which may predict a certain degree of neutralizing activity in the cohort. The rational cut-off value may help to predict the approximate humoral antiviral activity in individuals naturally infected with SARS-CoV-2.

## 2 Materials and methods

### 2.1 Study design and participants

**Fig. 1:**
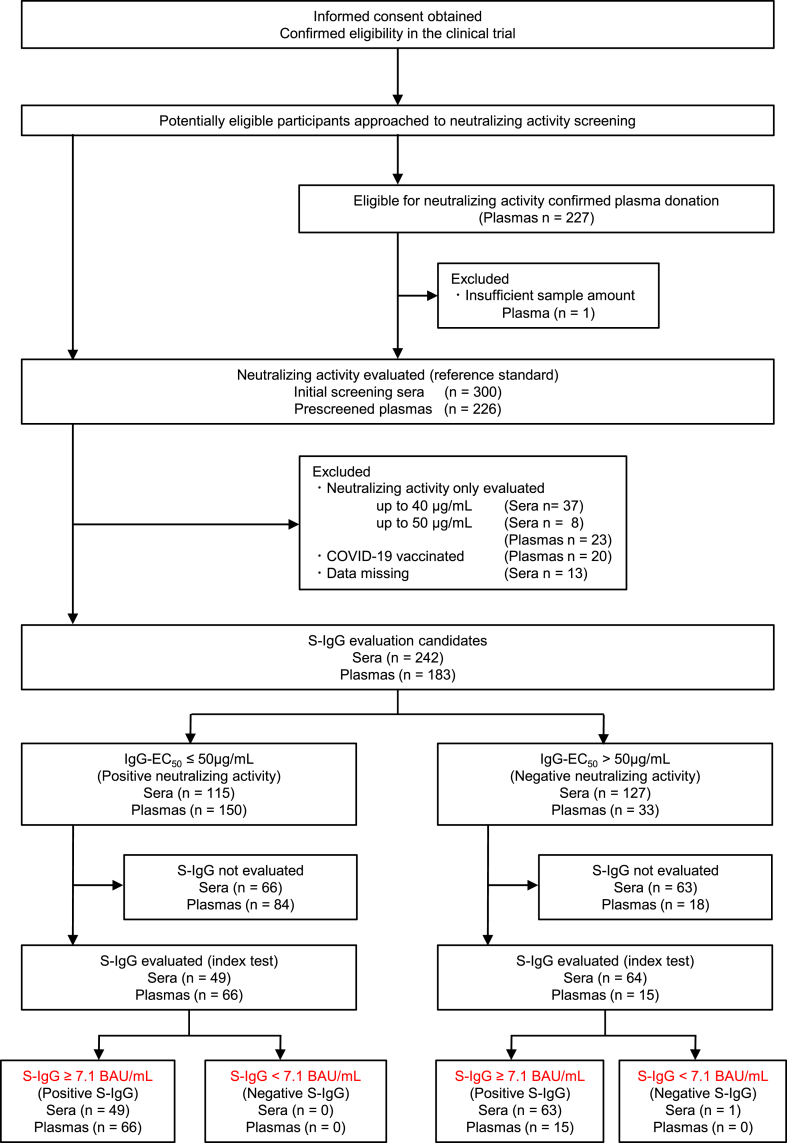
Flow diagram of the sera/plasma samples.

We conducted a cohort study to evaluate the presence of serum/plasma neutralizing activity in COVID-19 convalescent individuals in April 2020, as previously described (clinical trial approval number NCGM-G-003536) [12]. Detailed methods of this study are described in the Supplementary Materials. We retrospectively selected and enrolled consecutive individuals from April 30, 2020, to July 16, 2021 (Fig. 1 and Table 1). We first determined the neutralizing activity of serum/plasma-derived IgG-EC_50_ as the reference standard (Fig. 1 and S1A). We excluded some serum/plasma samples for which the IgG-EC_50_ neutralizing activity was below the detection limit of 100 μg/mL, those collected after COVID-19 vaccination, and those from patients with missing data (Fig. 1). We selected approximately 200 samples with an as even as possible distribution of the IgG-EC_50_ to assess the correlation between IgG-EC_50_ and S-IgG (Fig. 1, Fig. S1B, and Table S1). To validate the intra-assay deviation, high neutralizing activity-confirmed convalescent plasma D43-derived IgG [13] was used as a reference control. The neutralizing activity was similar to that of our previous report with a standard deviation of less than 10 %. This study was reviewed and approved by the Institutional Review Board of the National Center for Global Health and Medicine (clinical trial approval number: NCGM-G-003536). All participants provided informed consent to take part in the study.

**Table 1:** Characteristics of the study.

	All	Initial screening sera	prescreened plasmas	*p* value
Number of participants	370	300	166	
Number of samples	526	300	226	
Sample collection		April 30, 2020 to November 26, 2020	May 18, 2020 to August 16, 2021	
Age, median years (IQR)	46 (38–55)	45 (36–54)	52 (44–59)	<0.0001
Sex, N (%)				0.2045
Male	203 (54.9)	163 (54.3)	101 (60.8)	
Female	167 (45.1)	137 (45.7)	65 (39.2)	
Severity of the disease, N (%)				0.0003
Mild	173 (46.8)	139 (46.3)	58 (34.9)	
Moderate	104 (28.1)	92 (30.7)	42 (25.3)	
Severe	61 (16.5)	43 (14.3)	46 (27.7)	
Critical	18 (4.9)	12 (4.0)	16 (9.6)	
Data unavailable	14 (3.8)	14 (4.7)	4 (2.4)	
Sample collection PSO				<0.0001
median days (IQR)	74 (39–122)	56 (35–96)	111 (78–158)	

IQR; Interquartile range, PSO; Post symptom onset.

### 2.2 Neutralizing activity-determining assays (reference test) and quantification of S-IgG (index test)

The SARS-CoV-2-neutralizing activity of IgG-EC_50_ was determined, as previously described, by employing wild-type SARS-CoV-2 and transmembrane serine protease 2-overexpressing VeroE6 (VeroE6^TMPRSS2^) cells as reference [[13], [14], [15]]. The detailed methods for determining neutralizing activity and quantification of the S-IgG are described in the Supplementary Materials.

### 2.3 Statistical analysis

The correlation between IgG-EC_50_ and S-IgG was determined using the Spearman's rank correlation coefficient. The computed correlation coefficient was considered low, moderate, and high when the value was <0.4, 0.4–0.7, and >0.7, respectively, according to the Guilford's Rule of Thumb.

The censored regression analysis was performed to evaluate the relationship between S-IgG and IgG-EC50. The differences in IgG-EC_50_ and S-IgG between the initially screened serum and prescreened-plasma samples were compared using the Wilcoxon rank-sum test. The analyses were conducted using the logarithmic IgG-EC_50_ and S-IgG values. To assess the performance of S-IgG in estimating IgG-EC_50_, we plotted ROC curves to determine the rational cut-off value. The area under the ROC curve (AUC) and the confidence interval (CI) were generated via the bootstrap method (2000 replications). The *ROC* function in the *epi* package and the *roc* and *ci* functions in the *pROC* package were used for ROC curve analysis. A true-positive was indicated by IgG-EC_50_ ≤ 50 μg/mL and S-IgG ≥287.54 BAU/mL, meaning that the index test (S-IgG ELISA) correctly identified the presence of neutralizing IgG activity (IgG-EC_50_ ≤ 50 μg/mL). In contrast, a false positive was indicated by IgG-EC_50_ > 50 μg/mL and S-IgG ≥287.54 BAU/mL, meaning that while the index test (S-IgG test) indicate the presence of neutralizing IgG activity, it is not actually present.

The reproducibility of the results was compared using Cohen's kappa statistics. Categorical and continuous variables are presented as number (%) and median (interquartile range: IQR), respectively. Fisher's exact test for categorical values and Wilcoxon's rank sum test for continuous values were used to compare the IgG-EC_50_ and S-IgG groups. All *p* values were two-sided, and statistical significance was defined as *p* < 0.05. The vglm function in the vglm package was used for censored regression analysis. All statistical analyses were performed using R statistical software version 4.0.2.

## 3 Results

### 3.1 Study population

We retrospectively selected and enrolled 300 consecutive individuals from April 30, 2020, to November 26, 2020, and 167 consecutive individuals who donated convalescent plasma from May 18, 2020, to August 16, 2021. We obtained 300 serum and 227 plasma samples from 370 participants based on the inclusion criteria (Fig. 1). Fig. 1 illustrates the flow of serum/plasma samples in this study during the evaluation of S-IgG using the quantitative anti-SARS-CoV-2 S-IgG test (Fig. 1 and Table 1).

All the participants were Asian (Japanese) and 167 (45.1 %) were female. The median age was 46 years (IQR, 38–55) (Table 1). The number (ratio) of individuals with mild symptoms (no pneumonia), moderate symptoms (pneumonia with no oxygen requirement), severe symptoms (pneumonia requiring oxygen treatment without mechanical ventilation), critical symptoms (respiratory failure requiring mechanical ventilation, septic shock, and/or multiple organ dysfunction), and no clinical information were 173 (46.8 %), 104 (28.1 %), 61 (16.5 %), 18 (4.9 %), and 14 (3.8 %), respectively. The median duration from symptom onset to screening and plasma donation were 56 (IQR, 35–96) days and 111 (IQR, 78–158) days, respectively (Table 1).

### 3.2 COVID-19 convalescent individuals present a variety of neutralizing activities against wild-type SARS-CoV-2 with detectable anti-SARS-CoV-2 S-IgG antibodies

Some serum/plasma samples were excluded from S-IgG evaluation, as described in the study design, and 242 serum and 183 plasma samples were included (Fig. 1 and S1A). The neutralizing activity of the selected serum/plasma-derived IgG-EC_50_ against the virus was measured using geometric mean (GM) 50 % effective concentration (EC_50_, [GM-IgG-EC_50_]) ± the geometric standard deviations (SD) of 41.4 ± 1.5 and 23.7 ± 1.4 μg/mL, respectively (Fig. S1A). When an IgG-EC_50_ value of ≤50 μg/mL was set as the cut-off value, 115 serum (47.5 %) and 150 plasma samples (82.0 %) were observed to possess neutralizing activity (Fig. 1). Notably, the purified IgG-EC_50_ obtained from the individuals who had received the COVID-19 mRNA-vaccine (either BNT162b2 or mRNA-1273) after recovering from the disease presented significantly higher activity with GM-IgG-EC_50_ value ± the geometric SD of 4.8 ± 1.4 μg/mL (Fig. S1A).

**Fig. 2:**
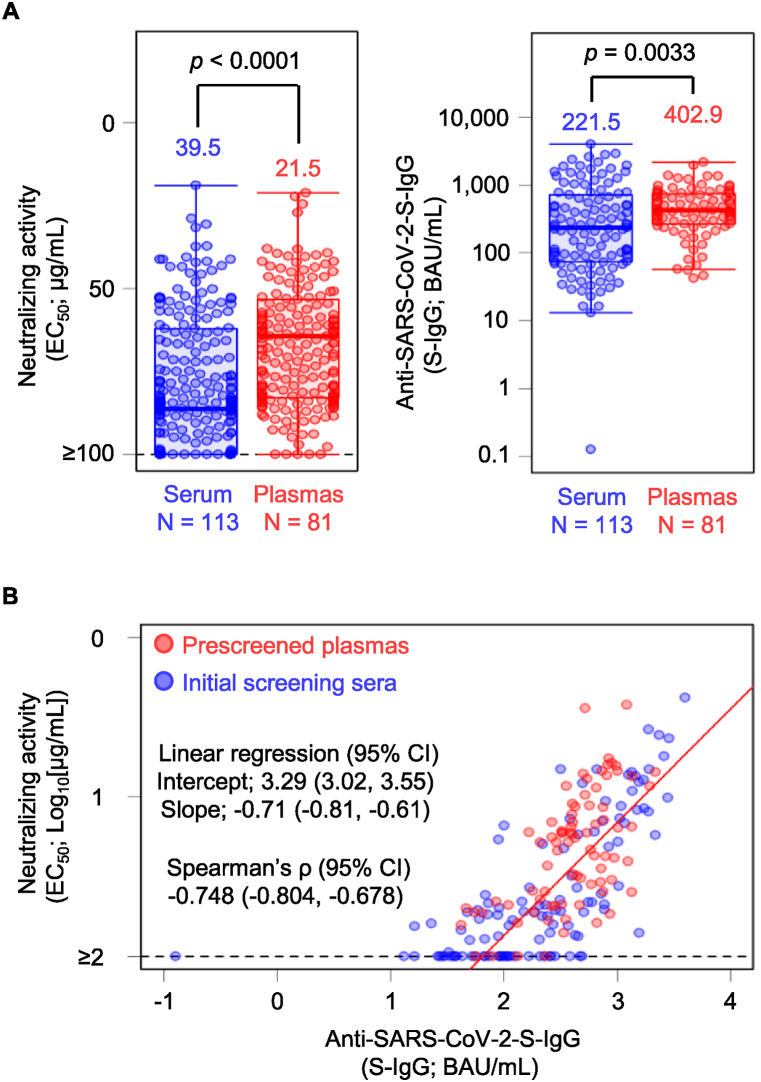
(**A**) Neutralizing activity of the purified IgG (IgG-EC_50_, left panel) and amount of the anti-SARS-CoV-2 Spike-IgG (S-IgG, right panel). The horizontal lines and black vertical bars represent geometric means (GM) and standard deviations (SD), respectively. The numbers over the markers indicate the GM values. The horizontal dashed lines indicate the limit of detection (100 μg/mL). As the plasma samples were collected from individuals who were confirmed to have a certain degree of neutralizing activity before donation, the plasma samples tested in the present study had significantly higher neutralizing activity and S-IgG levels compared with the serum samples, with *p* values < 0.0001 and 0.0033, respectively. (**B**) High correlation of IgG-EC_50_ with the amount of S-IgG with Spearman's ρ value of −0.748 (95 % CI, −0.804–0.678). One out of 113 serum samples had much lower amounts of S-IgG (0.9 BAU/mL) with no detectable neutralizing activity (IgG-EC_50_ > 100 μg/mL) and was excluded from the regression analysis. The censored regression analysis was performed to evaluate the relationship between S-IgG and IgG-EC_50_. IgG, immunoglobulin gamma; CI, confidence interval; SARS-CoV-2, severe acute respiratory syndrome coronavirus 2.

To evaluate the correlation and linear regression between IgG-EC_50_ and S-IgG, we randomly selected 113 serum samples (49 with IgG-EC_50_ ≤ 50 μg/mL) and 81 plasma samples (66 with IgG-EC_50_ ≤ 50 μg/mL) for even distribution of IgG-EC_50_ (Fig. 1, Fig. S1B, and Table S1). The GM-IgG-EC_50_ ± geometric SD of the S-IgG of the evaluated serum and plasma samples were 39.5 ± 1.5 and 21.5 ± 1.4 μg/mL, respectively (Fig. 2A and S1B), while the GM-S-IgG ± geometric SD were 221.5 ± 2.0 and 402.9 ± 1.4 binding antibody units (BAU)/mL, respectively (Fig. 2A). Notably, since the plasma samples used in this study were collected from individuals who had been confirmed to have developed a certain degree of neutralizing activity or quantity of S-IgG, the plasma samples had a significantly higher neutralizing activity (*p* < 0.0001) and larger S-IgG quantity (*p* = 0.0032) (Fig. 2A). When 7.1 BAU/mL was set as the cut-off value according to the manufacturer's instruction, 112 (49 with IgG-EC_50_ ≤ 50 μg/mL) serum (99.1 %) and 81 (66 with IgG-EC_50_ ≤ 50 μg/mL) plasma samples (100 %) were interpreted as seropositive (Fig. 1).

### 3.3 Purified IgG neutralizing activity highly correlates with the amount of anti-SARS-CoV-2 S-binding antibodies

We evaluated the direct correlation between IgG-EC_50_ and S-IgG using a linear regression analysis. As shown in Fig. 2B, consistent with previous data on serum neutralizing activity [7,16,17], IgG-EC_50_ highly correlated with S-IgG with a Spearman's ρ value of −0.748 (95 % CI, −0.804–0.678) (Fig. 2B). The censored regression analysis showed that the intercept and coefficient were 3.29 (95 % CI, 3.02–3.55) and −0.71 (95%CI, −0.81–0.61), respectively (Fig. 2B).

### 3.4 Quantitative anti-SARS-CoV-2 S-IgG antibody test can be used as a prediction tool for IgG neutralizing activity when the appropriate cut-off value is chosen

We further attempted to determine the sensitivity and specificity of the quantitative S-IgG antibody test to identify individuals who possess high neutralizing anti-SARS-CoV-2-IgG with rational cut-off values. The plasma samples used in this study were donated by pre-screened participants, which may cause selection bias during sampling. Therefore, we randomly selected 65 S-IgG-evaluated serum samples according to the distribution of the neutralizing activity of the original population, excluding individuals who had received the COVID-19 vaccine (Fig. 1 and S1C). The GM-IgG-EC_50_ ± geometric SD of the selected serum samples (n = 113) was 39.5 ± 2.7, which was not significantly different from that of the original IgG-EC_50_ of the evaluated population (n = 242, 41.4 ± 2.4) (Figs. S1A and B).

We have previously established and reported a screening system for the neutralizing activity of COVID-19 convalescent individuals’ sera/plasmas [12]. Among the 340 COVID-19 convalescent sera/plasmas evaluated, the median neutralizing activity was 52.4 μg/mL [13]. Therefore, we first selected a cut-off value of 50 μg/mL to predict approximately the top 50 % of humoral antiviral activity in individuals naturally infected with SARS-CoV-2. Similarly, cut-off values of 20 μg/mL and 10 μg/mL were selected to predict the top 20 % and 5 % humoral antiviral activities, respectively.

When the IgG-EC_50_ values of 50 μg/mL, 20 μg/mL, and 10 μg/mL—which were the best 55.7 %, 19.2 %, and 7.3 % of the 287 IgG-EC_50_-determined serum samples, respectively, excluding those of individuals who received COVID-19 vaccines—were set as positive neutralizing activity cut-off values, the number of positive IgG-EC_50_ serum samples were 49 (43.4 %), 32 (28.3 %), and 15 (13.3 %), respectively (Table 2a–c). These results were similar to those obtained for the original 242 serum IgG-EC_50_ described above. When the S-IgG value of 7.1 BAU/mL was set as the cut-off value, according to the manufacturer's instruction, the number of positive and negative antibody tests were 112 (99.1 %) and 1 (0.9 %), respectively (Table S3).

**Table 2:** Performance of anti-SARS-CoV-2 Spike-IgG antibody test to predict IgG-EC50 neutralizing activity level 2(a) IgG-EC_50_ cut-off value of 50 μg/mL."the 1st Table - Kappa; 0.6397 (95 % CI 0.4964, 0.7829)" 2(b) IgG-EC_50_ cut-off value of 20 μg/mL."the 2nd Table - Kappa 0.6524 (95 % CI 0.5001–0.8047)." 2(c) IgG-EC_50_ cut-off value of 10 μg/mL."the 3rd Table - Kappa 0.6546 (95 % CI 0.4503–0.8590)."

	Purified-IgG neutralizing activity (IgG-EC_50_ μg/mL)		
anti-SARS-CoV- 2Spike-IgG (S-IgG, BAU/mL)	≤50 (Positive) N (%)	>50 (Negative) N (%)	Total N (%)
≥287.54 (Positive)	39 (34.5)	10 (8.8)	49 (43.4)
<287.54 (Negative)	10 (8.8)	54 (47.8)	64 (56.6)
Total	49 (43.4)	64 (56.6)	113

	Purified-IgG neutralizing activity (IgG-EC_50_ μg/mL)		
anti-SARS-CoV-2 Spike-IgG (S-IgG, BAU/mL)	≤20 (Positive) N (%)	>20 (Negative) N (%)	Total N (%)
≥454.14 (Positive)	27 (23.9)	12 (10.6)	39 (34.5)
<454.14 (Negative)	5 (4.4)	69 (61.1)	74 (65.5)
Total	32 (28.3)	81 (71.7)	113

	Purified-IgG neutralizing activity (IgG-EC_50_ μg/mL)		
anti-SARS-CoV-2 Spike-IgG (S-IgG, BAU/mL)	≤10 (Positive) N (%)	>10 (Negative) N (%)	Total N (%)
≥1162.84 (Positive)	12 (10.6)	7 (6.2)	19 (16.8)
<1162.84 (Negative)	3 (2.7)	91 (80.5)	94 (83.2)
Total	15 (13.3)	98 (86.7)	113

CI, confidence interval; IgG, immunoglobulin gamma; SARS-CoV-2, severe acute respiratory syndrome coronavirus 2.

**Fig. 3:**
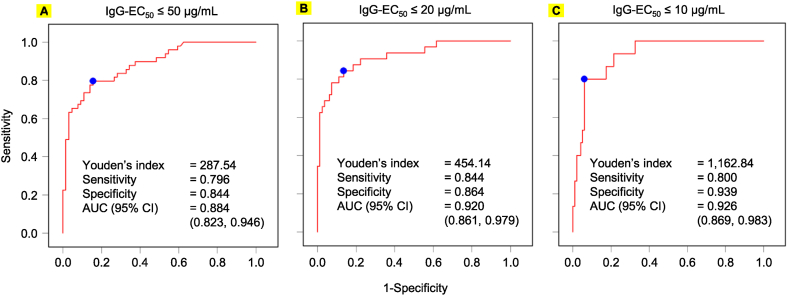
Receiver-operating characteristic (ROC) curve analysis using two different cut-off values. (**A**) ROC curve of 65 serum samples for moderate neutralizing activity. The prevalence of the neutralizing activity among serum samples with IgG-EC_50_ values ≤ 50 μg/mL in the analysis was 47.7 % (95 % CI, 35.1 %−60.5 %). This result was comparable with that for the 287 IgG-EC_50_-determined serum samples (52.5 %). The Youden's index was 287.5 BAU/mL with a sensitivity and specificity of 0.796 and 0.844, respectively, and an AUC of 0.884 (95 % CI, 0.823–0.946). (**B**) Screening of potent serum neutralizing activity with IgG-EC_50_ values ≤ 20 μg/mL, which represented approximately the best 18.5 % of the 287 IgG-EC_50_-determined serum samples. The cut-off value of 454.14 BAU/mL produced the highest sensitivity and specificity, at 0.844 and 0.864, respectively, with a AUC of 0.920 (95 % CI, 0.861–0.979) among the four analyses. AUC, area under the receiver operating characteristic curve; CI, confidence interval; IgG, immunoglobulin gamma.

As the S-IgG cut-off value of 7.1 BAU/mL was chosen to determine the presence of SARS-CoV-2 Spike protein-specific IgG antibodies and not to assess immunity against the pathogen, the cut-off value produced high sensitivity and extremely low specificity, making it a tool not worth more than a bare mention (Table S3). Therefore, we plotted a ROC curve to identify the appropriate cut-off value to estimate the IgG-EC_50_ with the S-IgG test. When 65 serum samples were analyzed based on the IgG-EC_50_ value of 50 μg/mL, the Youden's index [18] was 287.54 BAU/mL, with a sensitivity and specificity of 0.796 and 0.844, respectively, and an AUC value of 0.884 (95 % CI, 0.823–0.946) (Fig. 3A). When the IgG-EC_50_ value of 20 μg/mL was set as the cut-off value in the same population owing to its more potent neutralizing activity, the Youden's index was 454.14 BAU/mL, with a sensitivity and specificity of 0.844 and 0.864, respectively, and an AUC value of 0.920 (95 % CI, 0.861–0.979) (Fig. 3B).

These findings determined two cut-off values: moderate- (287.54 BAU/mL for the IgG-EC_50_ value of 50 μg/mL) and high-neutralizing activity (454.14 BAU/mL for the IgG-EC_50_ value of 20 μg/mL). As presented in Table 2a and S5, the positive predictive value (PPV), negative predictive value (NPV), positive likelihood ratio (LR+), and negative likelihood ratio (LR-) of the moderate-neutralizing activity were 0.80 (95 % CI, 0.66–0.90), 0.84 (95 % CI, 0.73–0.92), 5.09 (95 % CI, 2.83–9.16), and 0.24 (95 % CI, 0.14–0.43), respectively (Tables 2 and S5). The validity of the results was moderate with kappa statistics of 0.6397 (95 % CI, 0.4964–0.7829) [19]. Furthermore, as presented in Table 2b, the PPV, NPV, LR+, and LR-values for high-neutralizing activity were 0.69 (95 % CI, 0.52–0.83), 0.93 (95 % CI, 0.85–0.98), 5.70 (95 % CI, 3.31–9.80), and 0.18 (95 % CI, 0.08–0.41), respectively (Tables 2b and S5). The validity of the results was substantial with kappa statistics of 0.6524 (95 % CI, 0.5001–0.8047). Moreover, as presented in Table 2c, the PPV, NPV, LR+, and LR-values for high-neutralizing activity were 0.63 (95 % CI, 0.38–0.84), 0.97 (95 % CI, 0.91–0.99), 11.20 (95 % CI, 5.25–23.89), and 0.22 (95 % CI, 0.08–0.59), respectively (Tables 2b and S5). The validity of the results was substantial with kappa statistics of 0.6546 (95 % CI, 0.4503–0.8590).

The high LR+ and low LR-of the two cut-off values indicated that the quantitative anti-SARS-CoV-2 S-IgG antibody test provides substantial-to-strong evidence of the presence or absence of moderate-to-high neutralizing activity of the anti-SARS-CoV-2-IgG when rational cut-off values are used (Table S5). The measured antibodies correlate with the neutralizing activity of anti-SARS-CoV-2-IgG, and the neutralization assay can be a useful method in some clinically relevant situations.

## 4 Discussion

Serum antibody tests are often used in the diagnosis of infectious and autoimmune diseases or confirmation of acquired immunity against pathogens [20]; however, such tests do not necessarily predict the presence of neutralizing antibodies or protection from disease. On the other hand, although the presence of 10 milli-international units (IU)/mL of anti-hepatitis B virus-surface antibodies was arbitrarily chosen as a reference value [21], it was reportedly significantly associated with disease protection [22] even in immunocompromised individuals, such as people living with HIV/AIDS. Nevertheless, the widespread use of these antibody tests highly relies on the performance or characteristics of the tools and the pathogens involved, such that the current tests in use have received clinical use authorization from the FDA or other official audit organizations. Moreover, to date, only limited evidence has been established on the relationship between antibody titer, antibody neutralizing activity, and disease protection using real world data.

Regarding COVID-19, Guo et al. reported the clinical usefulness of anti-SARS-CoV-2-IgM serological tests in combination with quantitative RNA-PCR [23]. Dolscheid-Pommerich et al. assessed the relationship between S1-IgG levels and the neutralizing activity of serially-diluted sera and demonstrated that the quantitative enzyme-linked immunosorbent assay may be useful for predicting neutralization titers [24]. On the other hand, Criscuolo et al. reported a poor direct correlation between antibody titers and neutralizing activity levels [25]. These findings suggested that although the serum neutralizing activity highly relies on the amount and activity of the neutralizing IgG antibodies, other immune mediators may possibly contribute to the activity. In this study, we first demonstrated the high correlation between IgG-EC_50_ and S-IgG. This study excluded the COVID-19 mRNA-vaccinated group from the evaluation of cutoff values due to the small sample size and the significant neutralizing activity over natural infection. However, as previously reported, the high correlation between the amount of S-IgG and serum neutralizing activity [7] suggests that this study's findings can be applied to the general population.

Moreover, we demonstrated that rationally determined cut-off values in the S-IgG test could predict the presence or absence of neutralizing antibodies with high LR + or low LR- (Table 2a–c). As neither virus nor pseudo-virus neutralization tests (VNTs or pVNTs) have been authorized as clinical diagnostic tools, the competitive neutralization tests (cVNTs) are the only tools for determining the neutralizing activity of antibodies *in vitro* in clinical use in the United Sates [26]. However, cVNTs can only determine the inhibitory ability of antibodies in RBD‒Angiotensin-converting enzyme 2 interaction. Thus, in clinical settings, the determination of serum neutralizing activity or specific antibodies is quite challenging. This could be a major clinical problem, especially when the evaluation of whether immunocompromised individuals have acquired immunity against the pathogens is essential. Okamoto et al. reported that individuals with hematological malignancy, especially B-cell lymphoma, tend to have lower immunological response to COVID-19 vaccines even after 1 year of completing immunotherapy and/or chemotherapy, and that the amount of CD19^+^ lymphocytes is crucial for developing antibodies [27]. These individuals often experience fatal outcomes upon infection even after receiving two doses of the COVID-19 mRNA-vaccine [28], whereas the administration of therapeutic anti-SARS-CoV-2 monoclonal antibodies drastically increases both S-IgG and serum neutralizing antibody titers in such individuals and may improve disease prognosis [29]. The estimation of neutralizing activity in such patients using the quantitative S-IgG test should assist physicians in clinical decision-making for treatment. On the other hand, a case-controlled cohort study in Japan reported that serum neutralizing antibody titers were not decreased in individuals with breakthrough infection after they had received two doses of the COVID-19 mRNA-vaccine compared to that of the controls [30], suggesting that passive or elicited immunity is directly responsible for the protection or prevention of severe diseases. Further investigation may be needed to assess the relationship between S-IgG and protection from disease.

This study has some limitations. First, since the samples were obtained as part of an ongoing clinical trial to assess the neutralizing activity of convalescent plasma [10], more individuals with severe disease and higher S-IgG or neutralizing activity may have been included in this study compared to the general population. Second, not all collected samples underwent the index test (S-IgG assessment) to determine the reference IgG-EC_50_, which might result in potential selection bias. However, even though random exclusion was performed to select the S-IgG evaluation candidates (Figs. S1A–C), there were no statistical differences in IgG-EC_50_ distributions (Figs. S1B and S1C and Table S1) among the population. Third, in the present study, evaluation was conducted with only a wild-type variant assay, but ideally, several variants harboring multiple amino acid mutations, such as variants of concern (VOCs), were included. Since commercially available antibody tests or VNTs have been developed targeting the actual wild-type strain, further research targeting VOCs is needed to confirm the current study results. Nevertheless, our current study, which only focused on the wild-type strain, still holds practical value in clinical settings, as no commercially available kits specifically target such VOCs. We believe that this approach will allow us to make significant contributions to the field and potentially improve the patients’ outcomes.

## 5 Conclusions

Our findings suggest that S-IgG highly correlates with the neutralizing activity of serum anti-SARS-CoV-2-IgG and that the quantification of S-IgG could aid in the estimation of serum neutralizing activity much more conveniently than VNTs in clinical settings. Further evaluation is required in studies with a larger sample including individuals who received COVID-19 vaccines, immunocompromised patients, healthcare providers, and children or elderly people.

## Funding source

This work was supported by the 10.13039/100009619Japan Agency for Medical Research and Development (10.13039/100009619AMED) (grant numbers JP20fk0108160 and JP20fk0108502 to Ke.M. and JP20fk0108502, JP20fk0108257, and JP20fk0108510 to H.M.); 10.13039/501100003478MHLW Research on Emerging and Re-emerging Infectious Diseases and Immunization Program (grant number JPMH20HA1006 to Ke.M.); and Intramural Research Program of the Center for Cancer Research, 10.13039/100000054National Cancer Institute, National Institutes of Health (H.M.). Intramural Research Program of National Center for Global Health and Medicine (21A2007D to Y.T.)Abbott Japan LLC provided reagents for the quantitative anti-SARS-CoV-2 Spike-IgG antibody tests under a cooperative research and development agreement (N.I.). These funding sources were not involved in the data collection, analysis, and interpretation; the report writing; or the decision to submit the paper for publication.

## Ethical approval statement

This study was reviewed and approved by the Institutional Review Board of the National Center for Global Health and Medicine (clinical trial approval number: NCGM-G-003536). All participants provided informed consent to participate in the study.

## Data availability statement

All data generated or analyzed during this study are included in this published article and its supplementary information files.

## CRediT authorship contribution statement

**Noriko Iwamoto:** Writing – review & editing, Writing – original draft, Project administration, Methodology, Investigation, Funding acquisition, Data curation, Conceptualization. **Yuki Takamatsu:** Writing – review & editing, Writing – original draft, Project administration, Methodology, Investigation, Formal analysis, Data curation, Conceptualization. **Yusuke Asai:** Writing – review & editing, Visualization, Software, Investigation, Formal analysis, Conceptualization. **Kiyoto Tsuchiya:** Writing – review & editing, Data curation. **Kouki Matsuda:** Writing – review & editing, Data curation. **Yusuke Oshiro:** Writing – review & editing, Data curation. **Natsumi Inamura:** Investigation, Data curation. **Mari Terada:** Writing – review & editing, Investigation, Data curation. **Takeshi Nemoto:** Investigation, Data curation. **Moto Kimura:** Investigation. **Sho Saito:** Writing – review & editing, Data curation. **Shinichiro Morioka:** Writing – review & editing, Investigation, Data curation. **Maeda Kenji:** Supervision, Methodology, Investigation, Funding acquisition, Data curation. **Hiroaki Mitsuya:** Supervision, Funding acquisition. **Norio Ohmagari:** Supervision.

## Declaration of competing interest

The authors declare the following financial interests/personal relationships which may be considered as potential competing interests:Noriko Iwamoto reports a relationship with 10.13039/100009015Abbott Japan LLC. that includes: funding grants.
