# Supplementary material for: High diagnostic accuracy of quantitative SARS-CoV-2 spike-binding-IgG assay and correlation with in vitro viral neutralizing activity
Source: Heliyon. 2024 Jan 13;10(2):e24513. doi: 10.1016/j.heliyon.2024.e24513 (PMC10831606; doi:10.1016/j.heliyon.2024.e24513)
Supplement: Multimedia component 1 [file mmc1.docx]

**Supplemental text**

Supporting Material and Methods

***Study design and participants***

We designed and established a community-based cohort for a clinical trial study in the Center Hospital of the National Center for Global Health and Medicine (NCGM) in Tokyo, Japan (clinical trial approval number: NCGM-G-003536) [13]. Individuals aged from 20 to 69 years who were confirmed to have had SARS-CoV-2 infection more than 3 weeks before eligibility assessment were enrolled. A total of more than 1,300 COVID-19 convalescent participants were assessed at the NCGM by the end of November 2021. These participants were recruited within the NCGM and among the public via calls through the NCGM website, television programs, cooperating medical institutes, and social networking services [13]. Self-reported demographic data (race, birthday, and sex), date of symptom onset, severity of disease, and date of COVID-19 vaccination and manufactured vaccine (if applicable) were collected for statistical analysis. The sera collected from eligible participants underwent further pre-donation screening for anti-SARS-CoV-2-antibodies as well as neutralizing and other hematological and cardiovascular tests [13]. The plasmas were donated from participants who met thresholds for anti-SARS-CoV-2-antibodies and neutralizing activity [13].

***Cells, viruses, and IgG purification***

TMPRSS2-overexpressing VeroE6 (VeroE6^TMPRSS2^) cells (RRID: CVCL_YQ49) were obtained from the Japanese Collection of Research Bioresources (JCRB) Cell Bank (Osaka, Japan). VeroE6^TMPRSS2^ cells were maintained in Dulbecco’s modified Eagle’s medium (DMEM) supplemented with 10% fetal bovine serum, 100 μg/ml penicillin, 100 μg/ml kanamycin, and 1 mg/ml G418 under a humidified atmosphere containing 5% CO_2_ at 37 °C. The SARS-CoV-2 strain, SARS-CoV-2^05-2N^ (PANGO lineage B), was isolated in March 2020 in Tokyo, Japan as previously described [14]. IgG fractions were purified by using Spin column-based Antibody Purification Kit (Protein G) (Cosmo Bio, Tokyo, Japan). Donated sera/plasmas and purified-IgG were stored at -80 °C until use.

***Neutralizing activity-determining antiviral assays (reference standard) and quantification of the amount of S-IgG (index test)***

The SARS-CoV-2-neutralizing activity of purified IgG was determined as previously described, as a reference standard [14-16]. In brief, VeroE6^TMPRSS2^ cells were seeded in 96-well flat microtiter culture plates at the density of 1 × 10^4^ cells/well. On the following day, the virus (SARS-CoV-2^05-2N^) was mixed with various concentrations of the purified-IgG fractions and incubated for 20 min at 37 °C. The pre-incubated mixture was inoculated to the cells at a multiplicity of infection (MOI) of 0.01. The cells were cultured for 3 days and the number of viable cells in each well was measured using Cell Counting Kit-8 (Dojindo, Kumamoto, Japan). The potency of SARS-CoV-2 inhibition by purified IgG was determined based on its inhibitory effect on virally-induced cytopathicity in VeroE6^TMPRSS2^ cells. The amount of SARS-CoV-2 Spike-binding antibodies in each serum/plasma sample was determined by using ARCHITECT SARS-CoV-2 IgG II Quant (Abbott, Chicago, IL). The quantitative value in arbitrary unit (AU)/mL determined by the S-IgG test was converted to the binding antibody unit (BAU/mL), which is based on the WHO international standard anti-SARS-CoV-2 immunoglobulin (National Institute for Biological Standards and Control code: 20/136), as per the manufacturer’s instruction.

Supporting Figure Legends

Although the distribution of the IgG-EC_50_ of these randomly selected samples differed from the original neutralizing activity in the determined population (**Figures S1A and B**), there was no statistically significant difference in the variance of IgG-EC_50_ both in the sera and plasmas, with F values of 0.7734 (95% confidence interval [CI]; 0.5574–1.0534, *p* = 0.1034) and 0.8778 (95%CI; 0.5957–1.2584, *p* = 0.4742), respectively (**Figure S1B**).
