# Supplementary material for: High diagnostic accuracy of quantitative SARS-CoV-2 spike-binding-IgG assay and correlation with in vitro viral neutralizing activity
Source: Heliyon. 2024 Jan 13;10(2):e24513. doi: 10.1016/j.heliyon.2024.e24513 (PMC10831606; doi:10.1016/j.heliyon.2024.e24513)
Supplement: Multimedia component 2 [file mmc2.docx]

**Table S1. Characteristics of samples evaluated by anti-SARS-CoV-2 S-IgG antibody test**

|  | Initial screening  Serum | Prescreened  Plasma |  | *p* value |
| --- | --- | --- | --- | --- |
| Number of participants | 113 | 61 |  |  |
| Number of samples | 113 | 81 |  |  |
| Age, median years (IQR) | 44 (35-52) | 54 (45-60) |  | < 0.0001 |
| Sex |  |  |  | 0.6301 |
| Male | 63 (55.8) | 37 (60.7) |  |  |
| Female | 50 (44.2) | 24 (39.3) |  |  |
| Severity of disease, N (%) |  |  |  | 0.0027 |
| Mild | 51 (45.1) | 16 (26.2) |  |  |
| Moderate | 34 (30.1) | 13 (21.3) |  |  |
| Severe | 17 (15.0) | 24 (39.3) |  |  |
| Critical | 8 (7.1) | 7 (11.5) |  |  |
| Data unavailable | 3 (2.7) | 1 (1.6) |  |  |
| Sample collection PSO |  |  |  | < 0.0001 |
| median days (IQR) | 60 (35–123) | 130 (86–165) |  |  |

IQR: Interquartile range
