# Supplementary material for: High diagnostic accuracy of quantitative SARS-CoV-2 spike-binding-IgG assay and correlation with in vitro viral neutralizing activity
Source: Heliyon. 2024 Jan 13;10(2):e24513. doi: 10.1016/j.heliyon.2024.e24513 (PMC10831606; doi:10.1016/j.heliyon.2024.e24513)
Supplement: Multimedia component 3 [file mmc3.docx]

**Table S2.**  Comparison of IgG-EC50 distribution between all samples and S-IgG-measured samples using initial screening serum and prescreened plasma

| N (%) | Initial screening serum, N (%) | | *p* value |  | Prescreened plasma, N (%) | | *p* value |
| --- | --- | --- | --- | --- | --- | --- | --- |
| IgG-EC50 (μg/mL) | All candidates | S-IgG measured | 0.2212 |  | All candidates | S-IgG measured | 0.6216 |
| < 5 | 4 (1.7) | 4 (3.5) |  |  | 4 (2.2) | 2 (2.5) |  |
| 5 ≤ ~ < 10 | 15 (6.2) | 11 (9.7) |  |  | 24 (13.1) | 15 (18.5) |  |
| 10 ≤ ~ < 20 | 35 (14.5) | 17 (15.0) |  |  | 50 (27.3) | 24 (29.6) |  |
| 20 ≤ ~ < 50 | 61 (25.2) | 17 (15.0) |  |  | 72 (39.3) | 25 (30.9) |  |
| 50 ≤ ~ < 100 | 62 (25.6) | 29 (25.7) |  |  | 28 (15.3) | 11 (13.6) |  |
| 100 ≤ ~ | 65 (26.9) | 35 (31.0) |  |  | 5 (2.7) | 4 (4.9) |  |
