# Supplementary material for: High diagnostic accuracy of quantitative SARS-CoV-2 spike-binding-IgG assay and correlation with in vitro viral neutralizing activity
Source: Heliyon. 2024 Jan 13;10(2):e24513. doi: 10.1016/j.heliyon.2024.e24513 (PMC10831606; doi:10.1016/j.heliyon.2024.e24513)
Supplement: Multimedia component 4 [file mmc4.docx]

**Table S3. Accuracy of anti-SARS-CoV-2 S-IgG antibody test for neutralizing activity of initial-screening sera-derived purified-IgG with IgG-EC_50_ cut-off value of 50 μg/mL**

|  | Purified-IgG neutralizing activity  (IgG-EC_50_, μg/mL) | |  |
| --- | --- | --- | --- |
| anti-SARS-CoV-2 S-IgG  (S-IgG, BAU/mL) | ≤ 50 (Positive)  N (%) | > 50 (Negative)  N (%) | Total  N (%) |
| ≥ 7.1 (Positive) | 49 (43.4) | 63 (55.8) | 112 (99.1) |
| < 7.1 (Negative) | 0 (0) | 1 (0.9) | 1 (0.9) |
| Total | 49 (43.4) | 64 (56.6) | 113 |
