# Supplementary material for: High diagnostic accuracy of quantitative SARS-CoV-2 spike-binding-IgG assay and correlation with in vitro viral neutralizing activity
Source: Heliyon. 2024 Jan 13;10(2):e24513. doi: 10.1016/j.heliyon.2024.e24513 (PMC10831606; doi:10.1016/j.heliyon.2024.e24513)
Supplement: Multimedia component 6 [file mmc6.docx]

**Table S5. Summary of contingency tables with statistical values**

| Neutralizing activity cut-off |  | 50 μg/mL | 20 μg/mL |  | 50 μg/mL | 20 μg/mL | 10 μg/mL |
| --- | --- | --- | --- | --- | --- | --- | --- |
| S-IgG cut-off |  | 7.1 BAU/mL | |  | 287.54 BAU/mL (2,020 AU/mL) | 454.14 BAU/mL (3,200 AU/mL) | 1,162.84 BAU/mL  (8,190 AU/mL) |
| Contingency table |  | Table S3 | Table S4 |  | Table 2-a | Table 2-b | Table 2-c |
| Apparent prevalence  (95% CI) |  | 0.99  (0.95, 1.00) | 0.99  (0.95, 1.00) |  | 0.43  (0.34, 0.53) | 0.35  (0.26, 0.44) | 0.17  (0.10, 0.25) |
| True prevalence (95% CI) |  | 0.43  (0.34, 0.53) | 0.28  (0.20, 0.38) |  | 0.43  (0.34, 0.53) | 0.28  (0.20, 0.38) | 0.13  (0.08, 0.21) |
| Sensitivity  (95% CI) |  | 1.00  (0.89, 1.00) | 1.00  (0.84, 1.00) |  | 0.80  (0.66, 0.90) | 0.84  (0.67, 0.95) | 0.80  (0.52, 0.96) |
| Specificity  (95% CI) |  | 0.02  (0.00, 0.08) | 0.01  (0.00, 0.07) |  | 0.84  (0.73, 0.92) | 0.85  (0.76, 0.92) | 0.93  (0.86, 0.97) |
| Positive predictive value (PPV)  (95% CI) |  | 0.44  (0.34, 0.53) | 0.29  (0.20, 0.38) |  | 0.80  (0.66, 0.90) | 0.69  (0.52, 0.83) | 0.63  (0.38, 0.84) |
| Negative predictive value (NPV)  (95% CI) |  | 1.00  (0.01, 1.00) | 1.00  (0.01, 1.00) |  | 0.84  (0.73, 0.92) | 0.93  (0.85, 0.98) | 0.97  (0.91, 0.99) |
| Positive likelihood ratio (LR+)  (95% CI) |  | 1.02  (0.99, 1.05) | 1.01  (0.99, 1.04) |  | 5.09  (2.83, 9.16) | 5.70  (3.31, 9.80) | 11.20  (5.25, 23.89) |
| Negative likelihood ratio (LR-)  (95% CI) |  | 0.00  (0.00, NaN) | 0.00  (0.00, NaN) |  | 0.24  (0.14, 0.43) | 0.18  (0.08, 0.41) | 0.22  (0.08, 0.59) |

NaN; Not a number
