# Supplementary material for: High diagnostic accuracy of quantitative SARS-CoV-2 spike-binding-IgG assay and correlation with in vitro viral neutralizing activity
Source: Heliyon. 2024 Jan 13;10(2):e24513. doi: 10.1016/j.heliyon.2024.e24513 (PMC10831606; doi:10.1016/j.heliyon.2024.e24513)
Supplement: Multimedia component 7 [file mmc7.pptx]

## Slide 1
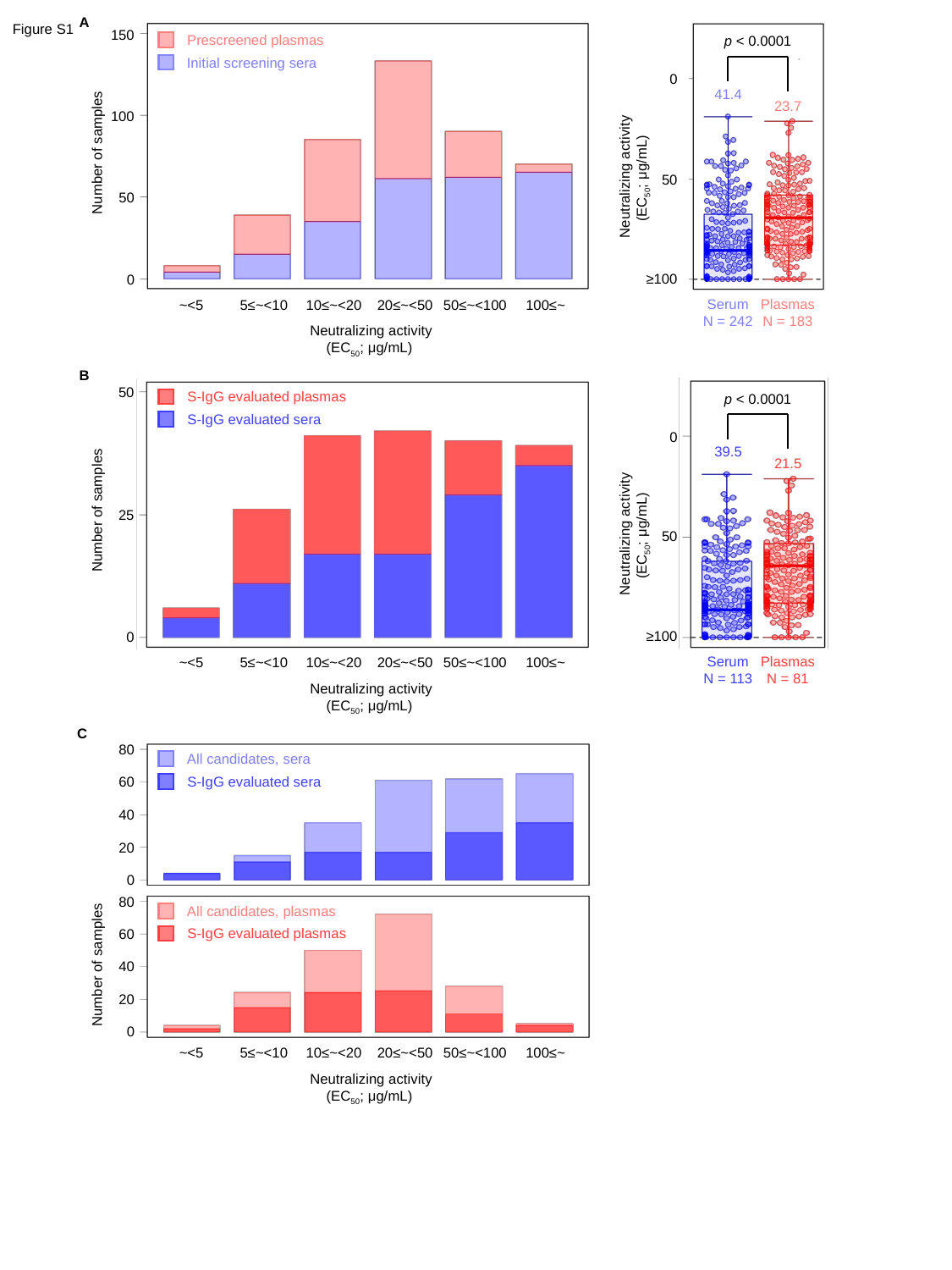

A
150
100
 Number of samples
50
0
Prescreened plasmas
Initial screening sera
~<5
 5≤~<10
 10≤~<20
 20≤~<50
 50≤~<100
 100≤~
 Neutralizing activity
(EC50; μg/mL)
p < 0.0001
41.4
23.7
0
 Neutralizing activity
(EC50; μg/mL)
50
≥100
Serum
N = 242
Plasmas
N = 183
Figure S1
 B
50
 Number of samples
25
0
p < 0.0001
39.5
21.5
0
 Neutralizing activity
(EC50; μg/mL)
50
≥100
Serum
N = 113
Plasmas
N = 81
S-IgG evaluated plasmas
S-IgG evaluated sera
~<5
 5≤~<10
 10≤~<20
 20≤~<50
 50≤~<100
 100≤~
 Neutralizing activity
(EC50; μg/mL)
C
80
60
40
20
0
80
60
40
20
0
All candidates, sera
S-IgG evaluated sera
All candidates, plasmas
S-IgG evaluated plasmas
 Number of samples
~<5
 5≤~<10
 10≤~<20
 20≤~<50
 50≤~<100
 100≤~
 Neutralizing activity
(EC50; μg/mL)
